# Population-based studies of myocardial hypertrophy: high resolution cardiovascular magnetic resonance atlases improve statistical power

**DOI:** 10.1186/1532-429X-16-16

**Published:** 2014-02-03

**Authors:** Antonio de Marvao, Timothy JW Dawes, Wenzhe Shi, Christopher Minas, Niall G Keenan, Tamara Diamond, Giuliana Durighel, Giovanni Montana, Daniel Rueckert, Stuart A Cook, Declan P O’Regan

**Affiliations:** 1 From the Medical Research Council Clinical Sciences Centre, Faculty of Medicine, Imperial College London, Hammersmith Hospital Campus, Du Cane Road, London W12 0NN, UK; 2 Department of Computing, Imperial College London, Kensington Campus, Exhibition Road, London SW7 2AZ, UK; 3 Department of Mathematics, Imperial College London, South Kensington Campus, Exhibition Road, London SW7 2AZ, UK; 4 Department of Cardiology, Imperial College NHS Healthcare Trust, Du Cane Road, London W12 0HS, UK; 5 Department of Cardiology, National Heart Centre Singapore, 17 Third Hospital Ave, Singapore 168752, Singapore; 6 Duke-NUS, 8 College Road, Singapore 169857, Singapore

**Keywords:** Imaging-genetics, LVH, Cardiomyopathy, GWAS, Biobank, Cardiovascular magnetic resonance, Image analysis

## Abstract

**Background:**

Cardiac phenotypes, such as left ventricular (LV) mass, demonstrate high heritability although most genes associated with these complex traits remain unidentified. Genome-wide association studies (GWAS) have relied on conventional 2D cardiovascular magnetic resonance (CMR) as the gold-standard for phenotyping. However this technique is insensitive to the regional variations in wall thickness which are often associated with left ventricular hypertrophy and require large cohorts to reach significance. Here we test whether automated cardiac phenotyping using high spatial resolution CMR atlases can achieve improved precision for mapping wall thickness in healthy populations and whether smaller sample sizes are required compared to conventional methods.

**Methods:**

LV short-axis cine images were acquired in 138 healthy volunteers using standard 2D imaging and 3D high spatial resolution CMR. A multi-atlas technique was used to segment and co-register each image. The agreement between methods for end-diastolic volume and mass was made using Bland-Altman analysis in 20 subjects. The 3D and 2D segmentations of the LV were compared to manual labeling by the proportion of concordant voxels (Dice coefficient) and the distances separating corresponding points. Parametric and nonparametric data were analysed with paired t-tests and Wilcoxon signed-rank test respectively. Voxelwise power calculations used the interstudy variances of wall thickness.

**Results:**

The 3D volumetric measurements showed no bias compared to 2D imaging. The segmented 3D images were more accurate than 2D images for defining the epicardium (Dice: 0.95 vs 0.93, P < 0.001; mean error 1.3 mm vs 2.2 mm, P < 0.001) and endocardium (Dice 0.95 vs 0.93, P < 0.001; mean error 1.1 mm vs 2.0 mm, P < 0.001). The 3D technique resulted in significant differences in wall thickness assessment at the base, septum and apex of the LV compared to 2D (P < 0.001). Fewer subjects were required for 3D imaging to detect a 1 mm difference in wall thickness (72 vs 56, P < 0.001).

**Conclusions:**

High spatial resolution CMR with automated phenotyping provides greater power for mapping wall thickness than conventional 2D imaging and enables a reduction in the sample size required for studies of environmental and genetic determinants of LV wall thickness.

## Background

The structure of the human heart is highly heritable [1] and influenced by complex interactions between multiple genes and environmental factors [2]. Left ventricular (LV) mass is a clinically important inheritable trait which independently predicts the risk of heart failure, sudden death and all-cause mortality [3], however the genes that control myocyte hypertrophy remain elusive [4]. Inherited cardiac conditions typically cause regional or asymmetric changes in LV structure and therefore total mass may be an insensitive indicator for detecting genetic influences. Quantitative phenotyping of the heart may overcome these limitations by creating detailed 3D statistical models of the variation in cardiac morphology and function within a population. A computational approach to phenotyping has been successfully used in brain mapping studies [5-9] and also has potential for epidemiological research in heart disease [10,11]. While neuroimaging benefits from high spatial resolution 3D magnetic resonance (MR) to detect anatomical variation, the statistical modelling of conventional 2D cardiac cine imaging is constrained by the low spatial resolution of each section and misalignment between breath-holds [12].

Standard 2D cine CMR comprises a stack of 8 mm sections with 2 mm gaps in the LV short axis plane which are acquired over several breath-holds [13]. Three dimensional cardiac imaging, at a comparable spatial resolution to 2D sequences, has been shown to have similar accuracy for manual assessment of LV volumes and mass [14-23]. To achieve 3D coverage of the whole heart at greater spatial resolution in a single breath-hold requires the use of high acceleration factors coupled with automated analysis techniques that enable accurate mapping of ventricular wall thickness. In this study we used a 3D cine sequence with a spatial resolution twice that of conventional 2D imaging and analysed the images by means of a set of 3D cardiac atlases to guide detection and co-registration of the myocardium. This approach enables the statistical variation in wall thickness to be mapped at corresponding points of the LV within a group of subjects.

The primary purpose of this study was to evaluate the feasibility and accuracy of high spatial resolution3D cine imaging for phenotypic analysis of the LV; and secondarily to determine if this would enable a reduction in the sample size required for population-based research of myocardial hypertrophy.

## Methods

The study was supported by the Medical Research Council, UK, the National Institute for Health Research (NIHR) Biomedical Research Centre based at Imperial College Healthcare NHS Trust and Imperial College London, UK, and a British Heart Foundation, UK, project grant (PG/12/27/29489) and special grant (SP/10/10/28431). The authors had control of the data and information submitted for publication.

### Study population

This single-center prospective study was approved by the Hospital’s research ethics committee and all participants gave written informed consent. In total 138 adult volunteers (78 females: age range 18–65 years; mean 39.9 years) were recruited via advertisement for a sub-study of the UK 1000 Cardiac Phenomes project. We excluded participants at screening that had known cardiovascular disease or were being treated for hypertension, diabetes or hypercholesterolemia. Female subjects were excluded if they were pregnant or breastfeeding but were eligible if they took oral contraceptives. Standard published safety contraindications to MR imaging were applied [24]. Twenty subjects were used to create the cardiac atlases (10 female, age range 24–59 years, mean 38.0 years), and 20 volunteers (9 female, age range 18–54 years, mean 36.5 years) were imaged on two separate occasions for assessment of reproducibility.

### MR imaging protocol

CMR was performed on a 1.5T Philips Achieva system (Best, Netherlands). The maximum gradient strength was 33 mT/m and the maximum slew rate 160 mT/m/ms. A 32 element cardiac phased-array coil was used for signal reception. Scout images were obtained and used to plan 2D cine balanced steady-state free precession (b-SSFP) images in the left ventricular short axis (LVSA) plane from base to apex using the following parameters: repetition time msec/echo time msec, 3.0/1.5; flip angle, 60°; bandwidth, 1250 Hz/pixel; acquired pixel size, 2.0 × 2.2 mm; section thickness 8 mm with a 2 mm gap; reconstructed voxel size, 1.2 × 1.2 × 8 mm; number of sections, 10 – 12; cardiac phases, 30. A single breath-hold 3D LVSA b-SSFP sequence was acquired in the same orientation using the following parameters: 3.0/1.5; flip angle, 50°; bandwidth, 1250 Hz/pixel; pixel size 2.0 × 2.0 mm; section thickness, 2 mm overlapping; reconstructed voxel size, 1.2 × 1.2 × 2 mm; number of sections, 50 – 60; cardiac phases, 20; sensitivity encoding (SENSE) factor 2.0 anterior-posterior and 2.0 right-left direction.

### Image segmentation

**Figure 1:**
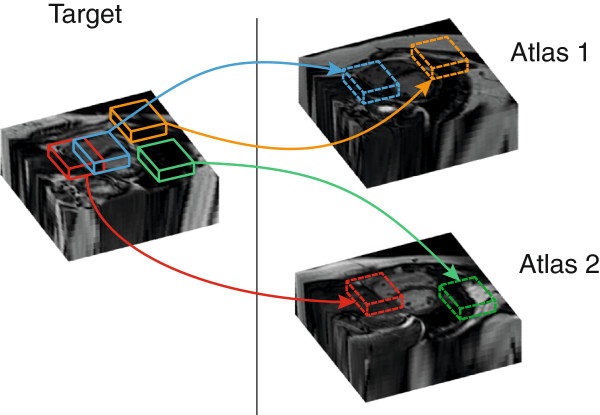
**The automated cardiac segmentation method looked for similarities between cubic "patches" in the manually-labeled pool of atlases and each new target image.** Correspondences with two atlases are shown in the diagram, but up to 20 high spatial resolution atlases were simultaneously used for accurate segmentation in the study.

To guide image segmentation 20 cardiac atlases were created to provide prior information about the inter-subject variability in cardiac anatomy [25]. A set of 3DLVSA images from 20 subjects had each voxel manually labeled as LV cavity, myocardium or right ventricle cavity on the end-diastolic image by two readers using freely available software (ITKsnap, National Library of Medicine’s Insight Segmentation and Registration Toolkit, http://www.itk.org) [26]. Subsequent image segmentation was automated but was initialised using six pre-defined landmarks on each target image. A multi-atlas PatchMatch algorithm [27] was used to find correspondences between "patches" of neighbouring voxels (5 × 5 × 5 mm) within the atlases and target images (Figure 1). Each selected atlas patch was given a weighting according to its similarity and distance to the target patch. Labels from all the atlas patches were then combined to produce a final segmentation. Lastly, the mean shape of all atlases in the pool was co-registered to the segmentation to ensure each spatial coordinate in the 3D model was consistent between all subjects. Endocardial and epicardial mesh surfaces, with 8122 and 10696 points respectively, were reconstructed using the marching cubes algorithm [28,29]. Analysis was performed using numerical computing software (Matlab, Natick, MA) on a workstation (Xeon quad-core 2.4 GHz with 8GB of random access memory; Intel, Santa Clara, CA).

All data were represented in a standard coordinate system and visualized on a 3D myocardial template created from the mean shape of the 20 atlases. Wall thickness was measured perpendicular to a midwall plane equidistant to the endocardial and epicardial surfaces. The volume of the voxels labeled as left ventricle cavity and myocardium were used to calculate LV end-diastolic volumes and mass which were then indexed to body surface area (LVEDVI and LVMI respectively). Myocardial density was assumed to be 1.05 g/mL [30].

To assess the accuracy of segmentation each of the 20 atlases was segmented using the other 19 and the results compared to its own manually labeled atlas. To enable an unbiased evaluation, the 2D LVSA images were compared to the corresponding manually-labeled atlas down-sampled to the equivalent spatial resolution. For the 3D and 2D LVSA data the distance between each epicardial and endocardial point on the segmentations and its corresponding point on the labeled atlas was measured. The accuracy of segmentation was also assessed by the proportion of concordant voxels in the target images and the corresponding manually labeled atlas. This agreement was expressed using the Dice similarity coefficient where 0 indicates no overlap and a value of 1 indicates perfect agreement [31].

### Reliability

Twenty subjects had 2D and 3D LVSA imaging performed on two separate occasions. In each case the subject briefly got off the MR table and the study was then repeated with new pilot images. An independent reader of 10 years cardiac MR experience manually analysed the 2D LVSA datasets using cardiac analysis software (Extended WorkSpace, Philips, Best, Netherlands). Endocardial and epicardial borders were defined on the left ventricular cine images using a standard methodology to derive LVEDVI and LVMI [32]. Trabeculae and papillary muscles were excluded from the mass measurement and included in the cavity volume.

### Image quality assessment

Two readers with 10 and 6 years of experience in CMR, who were blinded to the imaging protocol, independently assessed the image quality of the 2D and 3D LVSA images in 20 subjects. The overall image quality of each technique was assessed on a four-point scale (score 1, severe artefact or poor image quality making the myocardium not evaluable; score 2, fair delineation of the myocardium with moderate artefact affecting the heart; score 3, good delineation of the myocardium with mild artefact affecting the heart; and score 4, excellent delineation of the myocardium and no artefacts within the heart).

To account for spatial variation in image noise on the undersampled images the contrast on 2D and 3D cine images was assessed by measuring contrast ratios [33]. A contrast ratio (CR) was calculated using the following equation: $\text{CR}={\text{SI}}_{1}-{\text{SI}}_{2}/\surd \left({\text{SD}}_{1}^{2}+{\text{SD}}_{2}^{2}\right)$, where SI_1_ and SI_2_ are the mean signal intensities of relatively homogeneous areas of the myocardium and blood pool and SD_1_ and SD_2_ are their respective standard deviations.

### Statistical analysis

Data was analysed using R version 3.0.1 [34] and SigmaPlot (Systat Software, San Jose, CA). Normally distributed data were reported as mean ± one standard deviation (SD) or otherwise as median and interquartile range (IQR). Comparison between methods was made using Bland-Altman plots [35]. CRs were compared using a two-sided paired t-test and reported with 95% confidence interval (CI) for the difference of the mean. Image quality scores were compared with the Wilcoxon signed-rank test. Test-retest reliability was assessed using an intraclass correlation coefficient (ICC) with a two-way random model for absolute agreement [36]. Voxelwise comparisons between the 2D and 3D techniques were made using the Wilcoxon signed rank test. The sample size required for automated segmentation of 2D and 3D techniques to detect a 1 mm difference in wall thickness at each point across the myocardium was calculated from the voxelwise interstudy variances (see Appendix) [37]. A P value <0.05 was considered significant and Bonferroni correction was made for multiple comparisons in all voxelwise tests.

## Results

All of the 138 healthy volunteers successfully completed the imaging protocol and all datasets were used for analysis. Subject characteristics are shown in Table 1. The 2D LVSA data acquisition required 5 or 6 breath holds of 12 – 15 s each while the typical breath hold for the 3D LVSA cine typically lasted 20–25 s depending on heart rate and number of sections acquired.

**Table 1:** Subject characteristics and CMR derived cardiac measurements (n = 138)

	**Men (n = 60)**			**Female (n = 78)**		
	**Mean**	**SD**	**%**	**Mean**	**SD**	**%**
Age (yrs)	38.1	11.2		41.5	12.3	
Race/Ethnicity						
Caucasian			73.3			74.4
Asian Subcontinent			15			15.4
Afro Caribbean			3.3			7.7
Other			8.3			2.6
Height (cm)	176.6	8.2		163.4	6.2	
Weight (kg)	79.7	13.4		65.8	9.3	
Body Surface Area (m^2^)	2.0	0.2		1.7	0.1	
Systolic BP (mmHg)	127.1	12.3		121.4	18.3	
Diastolic BP (mmHg)	81.4	10.4		80.2	11.5	
LVEDVI (mls/m^2^)	85.2	13.0		75.0	11.6	
LVMI (g/m^2^)	70.6	11.5		53.4	9.2	

### Image quality

The mean CR between blood pool and myocardium was greater on 2D images compared to 3D 12.2 ±2.5 vs 8.7 ± 1.9 (95% CI: 2.2 – 4.9; P < 0.0001). The image quality ratings for 2D imaging were slightly higher than for 3D imaging (median 4.0 vs 3.5, P = 0.002), but all images were interpretable.

### Accuracy of the automated 3D segmentations

**Figure 2:**
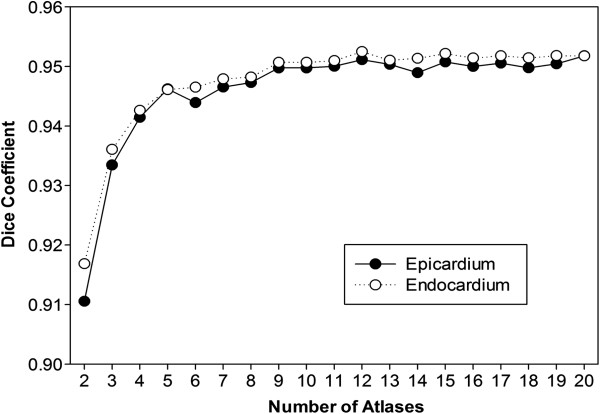
The accuracy of cardiac segmentation, measured with the Dice coefficient, improved as a larger number of 3D atlases were included in the analysis.

**Figure 3:**
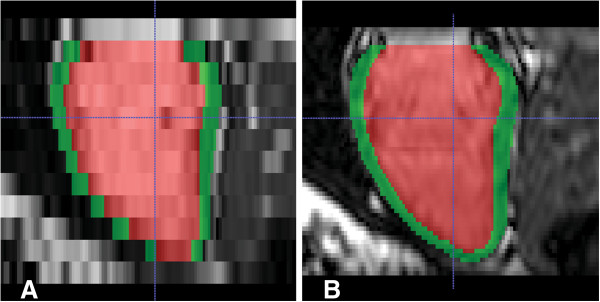
**Left ventricular long axis reconstruction of short-axis cine MR images at end-diastole in a healthy volunteer.** Automated segmentation of the myocardium is shown in green and the cavity in red. Data from a conventional 2D LVSA is shown in **A)** compared to a high-spatial resolution 3DLVSA in **B)**.

The accuracy of automated segmentation progressively improved as the number of cardiac atlases was increased, but there was modest benefit beyond 10 atlases (Figure 2).There was better segmentation accuracy of the 3D LVSA compared to 2D LVSA for both the endocardium, 0.952 vs 0.927 (P < 0.001) and epicardium, 0.952 vs 0.928 (P < 0.001) using the Dice overlap coefficients (Figure 3).

**Figure 4:**
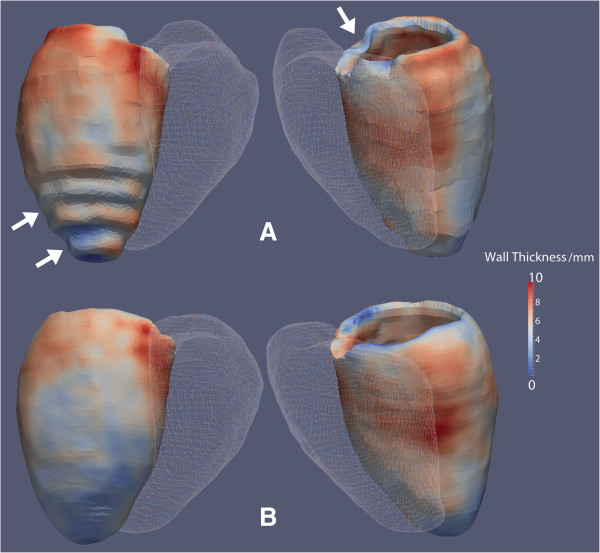
**Segmentations of the myocardium in a healthy volunteer with wall thickness shown as a color-scale.** Data from 2D **(A)** and 3D **(B)** LVSA cine images are presented with the right ventricle depicted as a mesh. Step artefact due to lower spatial resolution of 2D imaging is visible at the base and apex of the left ventricle (arrows).

The mean distance between the surfaces of the segmentation and the manually labeled reference was less for the 3D LVSA compared to the 2D LVSA for both endocardium: 1.09 mm ± 1.07 vs 2.02 mm ± 1.45 (95% CI: 0.74 mm – 1.12 mm; P < 0.001), and epicardium: 1.29 mm ± 1.32 vs 2.23 mm ± 1.64 (95% CI: 0.73 –1.14 mm; P < 0.001).An example of wall thickness mapping in one individual using 3D and 2D LVSA images is shown in Figure 4 where the effect of thinner sections using 3D imaging is most apparent at the base and apex of the LV.

**Figure 5:**
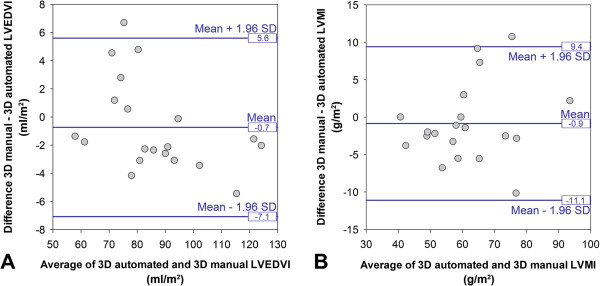
Comparison of the automated 3D LVSA segmentations to their respective manually labeled cardiac atlas in 20 volunteers demonstrating no bias for calculating LVEDI (A) or LVMI (B).

**Figure 6:**
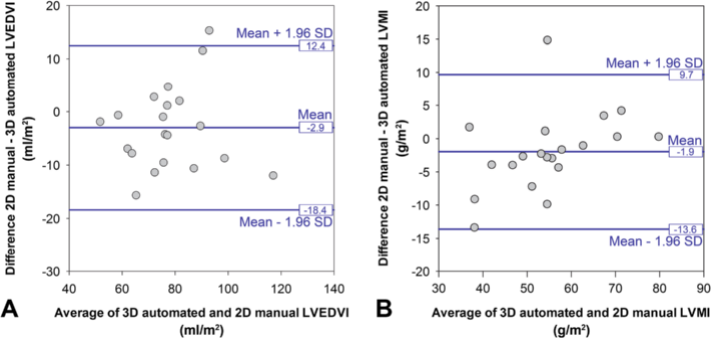
Comparison of the automated 3D LVSA segmentations to manual volumetry of the corresponding 2D LVSA images in 20 volunteers demonstrating no bias for calculating LVEDI (A) or LVMI (B).

Comparison of the 3D LVSA automated segmentations with the respective manually-labeled atlas showed that there was no bias introduced by the segmentation process for calculating LVEDI and LVMI (Figure 5). Comparison of the automated 3D segmentation with manual volumetry of the corresponding 2D images demonstrated that there was also no bias due to the different imaging techniques for calculating LVEDI and LVMI (Figure 6).

**Figure 7:**
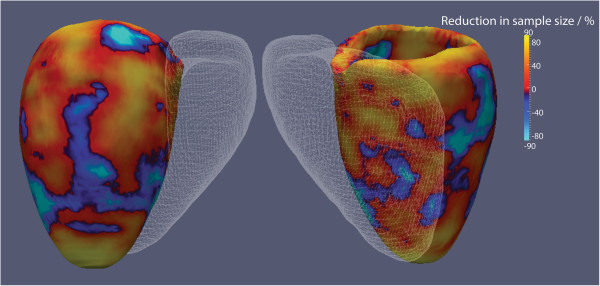
**The reduction in sample size required for 3D LVSA segmentations to detect a 1mm difference in left ventricular wall thickness compared to 2D LVSA segmentations is shown using reproducibility data from 20 healthy subjects.** The right ventricle is depicted as a mesh. The hot colors show where 3D imaging has the largest effect on reducing sample size which is predominantly at the basal and apical regions. (Values of ±90% are shown).

The power calculations, performed at each point on the co-registered myocardial surfaces, showed that overall fewer subjects were required for 3D imaging to detect a 1 mm difference in wall thickness than 2D imaging (56IQR: 39 – 78 vs 72 IQR: 49– 104, P < 0.001). The voxelwise reduction in sample size over the surface of the LV is shown in Figure 7.

### Reliability

The overall test-retest reliability of both 3D automated analysis and 2D manual analysis for global LV volumes and mass was high. The ICC between 3D LVSA segmentations was 0.97 (95% CI 0.93 – 0.99, P < .0001) for LVEDI and 0.93 (95% CI 0.82 – 0.97; P < .0001) for LVMI. The ICC between manual analyses of 2D LVSA images was 0.98 (95% CI 0.95 – 0.99; P < .0001) for LVEDVI and 0.97 (95% CI 0.91 – 0.99; P < .0001) for LVMI.

### Population mapping of wall thickness

**Figure 8:**
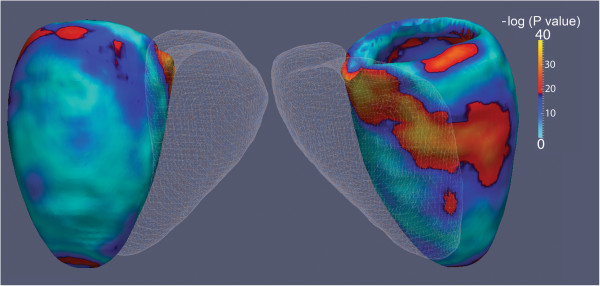
**Voxelwise comparisons between LV wall thickness on 2D and 3D LVSA myocardial segmentations in 100 healthy volunteers is shown.** The right ventricle is depicted as a mesh. A significance level of 5% corresponds to a value of 12.7 on the scale. Differences in wall thickness between the imaging techniques are apparent at highly curved regions of the base, interventricular septum and apex.

The images of 100 subjects were segmented with a mean unsupervised automated analysis time of 46 minutes per 3D sequence and 12 minutes for 2D. There were no failures of the segmentation algorithm in the cohort. Non-parametric comparison between 2D and 3D LVSA mapping of wall thickness is shown as a significance map in Figure 8.

## Discussion

The results of this study indicate that automated segmentation of high spatial resolution 3D cardiac imaging is a feasible technique for population phenotyping which provides greater accuracy for mapping ventricular wall thickness than conventional 2D cine MR. The greater statistical power to detect changes in wall thickness within the LV promises a significant reduction in sample size for epidemiological and genetic studies into the causes of myocardial hypertrophy and may also have applications in experimental medicine research and interventional trials.

Left ventricular hypertrophy (LVH) is an important cardiac phenotype which is associated with adverse cardiovascular outcomes. In order to explore the genetic and environmental determinants of LVH large populations are required to reach statistical significance when conventional CMR is used to measure total LV mass [38-40]. Although manual assessment of global LV parameters with CMR requires a smaller sample size to detect a given change in mass and volume than echocardiography [37], this approach does not extract the regional variations in ventricular wall thickness and function that are characteristic of both hypertrophic and hypertensive cardiomyopathies [41,42]. Cardiac atlases address this problem by creating computational models of phenotypic variation within a population by segmenting and co-registering each subject’s CMR images [10]. Previous studies have relied on conventional 2D CMR but its low through-plane spatial resolution sets intrinsic limitations on evaluating ventricular thickness and cardiac motion especially at the base and apex of the LV [43,44].

Three dimensional single breath-hold imaging of the LV has shown promise as a method to reduce total acquisition times and avoid section misalignment which provides good agreement for volumes and mass compared to 2D techniques [14-23]. These techniques have in general used parallel imaging or exploited temporal correlations in k-space to reduce acquisition time to a single breath hold. In our study we took advantage of the 3D acquisition to use SENSE in two spatial directions simultaneously [19] which significantly reduces geometry-related noise enhancement compared to 1-dimensional parallel imaging [45]. This enabled us to acquire high spatial resolution 3D cine images with an acceptable reduction in contrast and image quality compared to 2D imaging. Future studies in patient groups may also benefit from improvement in myocardium to blood pool contrast by using intravenous contrast medium [46].

As 3D imaging generates between 50 to 60 sections per cardiac phase manual analysis of the images is impractical and also does not fully exploit the advantages of whole organ imaging. Many approaches have been used for automated cardiac segmentation [12] and in our study we developed a multi-atlas technique which uses prior data from a set of manually-labeled high spatial resolution cardiac images. By finding correspondences between anatomical patches in the target images and each of the 20 twenty atlases we were able to achieve an accurate segmentation that was tolerant of lower image contrast and was not biased by any single atlas. We found a good agreement between our segmented data and conventional 2D volumetric quantification. However, the greatest advantage in 3D imaging was shown to be in assessing regional variations in wall thickness where partial volume effects at the base, septum and apex were significantly reduced compared to 2D imaging.

The ability to anatomically co-register each point in the heart throughout a large study population offers a powerful technique for extracting phenotypic data [10]. It allows the creation of statistical models of whole-heart physiology and anatomy that can be adjusted for anthropometric or environmental covariates. Neuroimaging studies have modelled the statistical power of 3D imaging [47] and our calculations indicate a significant reduction in sample size is possible over most of the myocardium for detecting differences in wall thickness. The primary application of this technique will be to allow prospective imaging-genetics association studies to be conducted more efficiently and reach statistical significance with fewer patients. Studies such as the 1000 Cardiac Phenomes project and the enhanced phase of UK Biobank will collect cardiac MR data from large cohorts of unselected participants and these techniques will enable comprehensive, efficient and statistically powerful computational modelling of the biological effects of environment and genetic effects on cardiac structure and function [48]. The integration of high-resolution imaging with multi-parametric "-omics" data may also have a role in "precision medicine" where diagnostic, prognostic, and therapeutic strategies are specifically tailored to each patient’s requirements [49].

Our study had limitations. We did not include patients with cardiovascular disease in this study and so we do not know if the imaging sequence or segmentation methods will be transferable to all patient groups. Our power calculations were conservative and did not model the more plausible biological effects of concentric or localised asymmetric changes in wall thickness. The pool of 20 atlases is relatively small compared to brain studies and may under-represent more extreme phenotypes. Translating these approaches to genetic studies will require the development of regression models which address the potential problem of multiple testing within large imaging datasets. Although we acquired cine images throughout the cardiac cycle in both ventricles we did not assess ejection fraction or cardiac motion, nor did we analyse the right ventricle.

## Conclusions

In conclusion, this study of healthy adults demonstrated that automated segmentation of high spatial resolution single breath-hold 3D cine MR imaging is more accurate than conventional 2D imaging for mapping LV anatomy and offers a reduction in the sample size required for epidemiological and genetic studies of heart disease.

## Appendix

For simplicity we consider each of the *P* = 16386 points across the myocardium independently and assume that the difference in wall thickness is normally distributed. This allows us to compute the sample size required to detect a change in wall thickness of δ at point *j* given a significance level α, power p and variance $\sigma_{j}^{2}$ as

$$N_{j}=\frac{2f\left(\alpha ,p\right)\sigma_{j}^{2}}{\delta^{2}}$$

where *f*(*α*, *p*) = (*u*_*α*_ + *u*_2(1 - *p*)_) where *u*_*γ*_ is the value of the standard Normal distribution (mean 0 and variance 1) such that the probability of lying between - *u*_*γ*_ and *u*_*γ*_ is 1 – γ [50]. Here, *α* and *p* can be set accordingly; typical values are *α* = 0.05 and *p*∈ [0.8, 0.9], and here we use *p* = 0.9. In order to conservatively account for the multiple comparisons in considering all *P* points independently, we use the Bonferroni adjusted significance level of *α/P* instead of simply *α*. Then *f*(*α*/*P*, *p*) = 35.3892.

Two myocardium images were obtained for each of *N* = 20 control subjects at the two time-points; 2D segmentations denoted $\left\{x_{\text{ij}}^{2D}\right\}$ and $\left\{y_{\text{ij}}^{2D}\right\}$, and 3D segmentations denoted $\left\{x_{\text{ij}}^{3D}\right\}$ and $\left\{y_{\text{ij}}^{3D}\right\}$ for *i =1,…,N* and *j = 1,…,P.* For the 2D and 3D segmentations, the differences in wall thickness between time-points at each point across the myocardium are given by $\left\{d_{\text{ij}}^{2D}\right\}=\left\{x_{\text{ij}}^{2D}-y_{\text{ij}}^{2D}\right\}$ and $\left\{d_{\text{ij}}^{3D}\right\}=\left\{x_{\text{ij}}^{3D}-y_{\text{ij}}^{3D}\right\}$ respectively. We note that for both the 2D and 3Dsegmentations, the differences do not exhibit non-normal behaviour (Shapiro test p-values are greater than the Bonferroni adjusted 5% significance level of 0.05/16386). Hence the assumption of normality of the differences which is required to use the above sample size formula is valid.

The variance of the differences at each of the *P* points of the 2D and 3D segmentations are computed and denoted ${\left(\sigma^{2}\right)}_{j}^{2D}$ and ${\left(\sigma^{2}\right)}_{j}^{3D}$. These can then be inserted in place of $\sigma_{j}^{2}$ in the above sample size formula. In particular, the sample size required to detect a change in wall thickness of *δ* = 1 at point *j* with *f*(*α*/*P*, *p*) = 35.3892 (*α* = 0.05/16386 and *p* = 0.9) for the 2D and 3D segmentations can then be obtained via

$$\begin{array}{ll} \;N_{j}^{2D} & =70.7784\times {\left(\sigma^{2}\right)}_{j}^{2D}\;\text{and}\;N_{j}^{3D}\\ & =70.7784\times {\left(\sigma^{2}\right)}_{j}^{3D} \end{array}$$

respectively. The percentage decrease in sample size in using a 3D segmentation over a 2D segmentation can be computed at each point as

$$\left(\frac{N_{j}^{2D}-N_{j}^{3D}}{N_{j}^{2D}}\right)\times 100$$

## Competing interests

The authors declare that they have no competing interests.

## Authors’ contributions

AdeM participated in the creation of the cardiac atlases, data analysis and drafted the manuscript. TJWD participated in the creation of the cardiac atlases and data analysis. WS and DR developed the co-registration and image analysis methods. CM and GM contributed to the statistical analysis of the data. GD scanned the patients and assisted in sequence development. TD recruited the subjects and collected anthrompometric data. NGK contributed to the manual data analysis. SAC and DPO’ Rconceived the research programme and coordinated this study. DPO’R participated in the drafting of the manuscript. All authors read and approved the final manuscript.
